# Impact of *CYP3A5*, *POR,* and *CYP2C19* Polymorphisms on Trough Concentration to Dose Ratio of Tacrolimus in Allogeneic Hematopoietic Stem Cell Transplantation

**DOI:** 10.3390/ijms20102413

**Published:** 2019-05-15

**Authors:** Kimitaka Suetsugu, Yasuo Mori, Nanae Yamamoto, Tomohiro Shigematsu, Toshihiro Miyamoto, Nobuaki Egashira, Koichi Akashi, Satohiro Masuda

**Affiliations:** 1 Department of Pharmacy, Kyushu University Hospital, 3-1-1 Maidashi, Higashi-ku, Fukuoka 812-8582, Japan; suetsugu@pharm.med.kyushu-u.ac.jp (K.S.); nyamamot@pharm.med.kyushu-u.ac.jp (N.Y.); shige825@pharm.med.kyushu-u.ac.jp (T.S.); n-egashi@pharm.med.kyushu-u.ac.jp (N.E.); 2 Department of Clinical Pharmacology and Biopharmaceutics, Graduate School of Pharmaceutical Sciences, Kyushu University, 3-1-1 Maidashi, Higashi-ku, Fukuoka 812-8582, Japan; 3 Department of Medicine and Biosystemic Science, Kyushu University Graduate School of Medical Sciences, 3-1-1 Maidashi, Higashi-ku, Fukuoka 812-8582, Japan; yasuomr@intmed1.med.kyushu-u.ac.jp (Y.M.); toshmiya@intmed1.med.kyushu-u.ac.jp (T.M.); akashi@med.kyushu-u.ac.jp (K.A.)

**Keywords:** *CYP3A5*, *POR*, *CYP2C19*, single nucleotide polymorphism, tacrolimus, hematopoietic stem cell transplantation

## Abstract

Single nucleotide polymorphisms in drug-metabolizing genes may affect tacrolimus pharmacokinetics. Here, we investigated the influence of genotypes of *CYP3A5*, *CYP2C19*, and *POR* on the concentration/dose (C/D) ratio of tacrolimus and episodes of acute graft-versus-host disease (GVHD) in Japanese recipients of allogeneic hematopoietic stem cell transplantation (HSCT). Thirty-six patients receiving the first HSCT using tacrolimus-based GVHD prophylaxis were enrolled with written informed consent. During continuous intravenous infusion, HSCT recipients carrying the CYP3A5*1 allele, particularly those with at least one POR*28 allele, had a significantly lower tacrolimus C/D ratio throughout all three post-HSCT weeks compared to that in recipients with POR*1/*1 (*p* < 0.05). The CYP3A5*3/*3 genotype and the concomitant use of voriconazole were independent predictors of an increased tacrolimus C/D ratio during the switch from continuous intravenous infusion to oral administration (*p* < 0.05). In recipients receiving concomitant administration of voriconazole, our results suggest an impact of not only *CYP3A5* and *CYP2C19* genotypes, but also plasma voriconazole concentration. Although switching from intravenous to oral administration at a ratio of 1:5 was seemingly appropriate in recipients with CYP3A5*1, a lower conversion ratio (1:2–3) was appropriate in recipients with CYP3A5*3/*3. Our results suggest that *CYP3A5*, *POR*, and *CYP2C19* polymorphisms are useful biomarkers for individualized dosage adjustment of tacrolimus in HSCT recipients.

## 1. Introduction

Tacrolimus has been widely used as an immunosuppressive drug for prophylaxis of graft-versus-host disease (GVHD) after allogenic hematopoietic stem cell transplantation (HSCT) [1,2,3,4,5,6]. GVHD remains the major cause of morbidity and mortality in recipients after HSCT; therefore, prevention of severe GVHD is crucial for the successful treatment of GVHD [7]. Given the narrow therapeutic range and the large inter- and intra-individual variabilities in the pharmacokinetics of tacrolimus [8], its blood concentration should be maintained at adequate levels to prevent drug-related toxicities. Tacrolimus is administered intravenously in the early phase after HSCT, followed by switching to an oral formulation when tolerated by recipients.

Single nucleotide polymorphisms (SNPs) in the genes encoding drug-metabolizing enzymes are considered to affect the pharmacokinetics of tacrolimus. Cytochrome P450 3A4 (CYP3A4) and CYP3A5 contribute to inter-individual variability in the metabolism of tacrolimus. Moreover, CYP3A5 may dominate CYP3A4 in the metabolism of tacrolimus in individuals expressing the CYP3A5 enzyme [9,10,11,12,13]. The most important SNP related to functional variation is CYP3A5*3 (6986A > G, rs776746), which causes abnormal mRNA splicing, resulting in a non-functional CYP3A5 protein [14,15]. Racial differences in the frequencies of *CYP3A5* polymorphisms are well acknowledged. The frequencies of CYP3A5*3/*3 have been reported to be 65%–73% in Asians, 87%–95% in Caucasians, and 27%–50% in the African-American population [16]. The majority of previous reports on organ transplant recipients show a significant effect of CYP3A5*3 on the pharmacokinetics of tacrolimus [17]. In HSCT recipients, several studies have shown that the concentration/dose (C/D) ratio or trough-blood concentration of tacrolimus is higher in recipients with the CYP3A5*3/*3 genotype than in those with the CYP3A5*1 allele (*1/*1 and *1/*3 genotypes) and that the required daily dosage of tacrolimus is, thus, significantly reduced [18,19,20,21]. However, reports discussing the utility of information related to *CYP3A5* polymorphism at the time of continuous intravenous infusion [18,19,20] and oral administration [21] are limited. Therefore, the effect of *CYP3A5* polymorphism on the pharmacokinetics of tacrolimus when switching from continuous intravenous infusion to oral administration is unclear. In contrast, although there are many genetic variants of *CYP3A4*, the majority of studies have failed to find an association between the *CYP3A4* genotype and tacrolimus pharmacokinetics [10,18,20]. Similarly, many studies suggest that SNPs in the ATP-binding cassette subfamily B member 1 transporter (*ABCB1*) gene do not influence tacrolimus pharmacokinetics, especially in Asian populations [10,18,20,22].

Genes located outside of the *CYP3A* locus may also influence the CYP3A phenotype. Cytochrome P450 oxidoreductase (POR) has recently been recognized as a potential contributor to intra- and inter-individual variability and an influencer of CYP3A activity. POR is involved in electron transfer from NADPH to the microsomal CYP enzymes, including members of the CYP3A subfamily, enabling their activities [23,24]. Human POR is highly polymorphic, and the most common sequence variant POR*28 (1508C > T; rs1057868) induces an amino acid substitution (Ala503Val) [25]. This substitution influences the electron-binding moiety of POR. The POR*28 SNP varies in frequency: 36% in Chinese-Americans, 26.4% in Caucasians, 19.1% in African-Americans, and 31% in Mexicans [25]. Those with the CYP3A5*1 allele carrying one or two POR*28 alleles (*1/*28 and *28/*28 genotypes) have lower tacrolimus C/D ratios and higher tacrolimus dose requirements than those with the CYP3A5*1 allele without POR*28 (*1/*1 genotype) among kidney transplant recipients [26]. However, it is still unclear whether the POR*28 polymorphism affects the pharmacokinetics of tacrolimus in HSCT recipients, as there are no reports describing this in HSCT.

When performing HSCT, azole antifungal agents such as fluconazole (FLCZ) and itraconazole (ITCZ) are most often used for the prevention or treatment of fungal infection [27,28]. The azole antifungal agents primarily inhibit the CYP3A4 enzyme [29,30,31]. Therefore, avoiding drug–drug interactions between tacrolimus and azole antifungal agents is difficult in HSCT, and differences in *CYP3A5* genotype may affect the interactions between these drugs. In addition, voriconazole (VRCZ), another azole antifungal agent, is metabolized by CYP2C19 as well as by CYP3A [32]. In particular, 15%–20% of Asians and 3%–5% of whites and blacks are estimated to be poor metabolizers of CYP2C19 [33]. Imamura et al. [34] reported that *CYP2C19* polymorphism is one of the key factors affecting the pharmacokinetics of tacrolimus in the concomitant administration of VRCZ in healthy Japanese volunteers. Therefore, the degree of drug interaction between tacrolimus and VRCZ may be influenced by *CYP2C19* and *CYP3A5* genotypes.

Accordingly, if information regarding *CYP3A5*, *POR*, and *CYP2C19* polymorphisms can be obtained prior to administration of tacrolimus, it would enable the adjustment of the initial dosage of tacrolimus and a reduction in the risk of adverse reactions. However, the clinical usefulness of such genetic polymorphism information in HSCT recipients has not yet been fully elucidated. In the present study, we examined the effect of gene polymorphisms on the pharmacokinetics of tacrolimus during the early stage of continuous intravenous infusion and when switching from continuous intravenous infusion to oral administration in HSCT recipients. We focused on the *CYP3A5*, *POR*, and *CYP2C19* polymorphisms and the interactions of tacrolimus with azole antifungal agents.

## 2. Results

### 2.1. Patient Background

The characteristics of 36 HSCT recipients according to their *CYP3A5* genotype are shown in Table 1. Nineteen recipients had at least one CYP3A5*1 allele, and 17 recipients had the CYP3A5*3/*3 genotype. There were no significant differences between the two groups with respect to age, sex, body weight, *POR* genotype, *CYP2C19* genotype, diagnosis, donor type, stem cell source, human leukocyte antigen (HLA) disparity, conditioning regimen, GVHD prophylaxis, concomitant antifungal agents, or levels of aspartate aminotransferase (AST), alanine aminotransferase (ALT), total bilirubin (T-Bil), or Scr.

### 2.2. Influence of CYP3A5*3 Genotype on C/D Ratio of Tacrolimus during Continuous Intravenous Infusion in the First Three Weeks following HSCT

We examined whether *CYP3A5* polymorphism influenced the C/D ratio of tacrolimus during continuous intravenous infusion in the first three weeks after HSCT. The median C/D ratios for each week after HSCT were calculated. Recipients with CYP3A5*3/*3 exhibited significantly higher median C/D ratios than those with the CYP3A5*1 allele during post-HSCT days 1–14 (post-HSCT days 1–7: *p* = 0.032; post-HSCT days 8–14: *p* = 0.001). However, there was no significant difference between the two groups during post-HSCT days 15–21 period (*p* = 0.860) (Table 2).

The influence of *CYP3A5* polymorphism on the tacrolimus C/D ratio without concomitant azole antifungal agent use was examined during the first three weeks. The only significant difference observed between the two groups was found at post-HSCT days 8–14 (*p* = 0.048) (Table 2).

### 2.3. Influence of the Combination of POR*28 and CYP3A5*3 Genotypes on Tacrolimus C/D Ratio during Continuous Intravenous Infusion in the First Three Weeks following HSCT

We examined whether *POR* polymorphism influenced the C/D ratio of tacrolimus in recipients carrying the CYP3A5*1 allele and CYP3A5*3/*3 genotype during continuous intravenous infusion in the first three weeks following HSCT. CYP3A5*1 allele carriers with one or two POR*28 alleles exhibited lower tacrolimus C/D ratios than CYP3A5*1 allele carriers with no POR*28 alleles throughout all three post-HSCT weeks (Table 3). In contrast, a significant difference between POR*28 allele carriers and non-carriers among those with the CYP3A5*3/*3 genotype was only observed at post-HSCT days 1–7 (*p* = 0.003) (Table 4).

The influence of the combination of *POR* and *CYP3A5* genotypes on the tacrolimus C/D ratio without concomitant azole antifungal agents was also examined. The POR*28 allele and CYP3A5*1 allele combination was associated with a significantly lower C/D ratio than the POR*1*/1 and CYP3A5*1 combination for the first and third weeks post-HSCT (post-HSCT days 1–7: *p* = 0.001; post-HSCT days 15–21: *p* < 0.001) (Table 3). In contrast, for those with the CYP3A5*3/*3 genotype, there was no significant association between the C/D ratio and *POR* polymorphism for any of the assessment periods (Table 4).

### 2.4. Relationship between CYP3A5*3 and Acute GVHD and AKI during the First Four Weeks following HSCT

**Figure 1:**
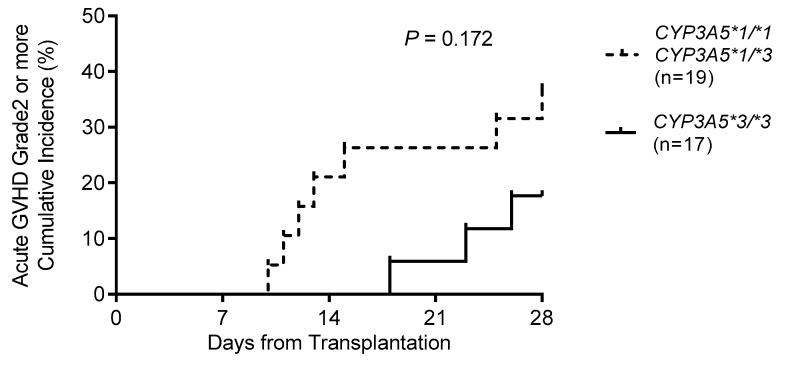
Cumulative incidence of grade II–IV acute graft-versus-host disease (GVHD) according to *CYP3A5* genotype during the first four weeks following HSCT. Cumulative incidence is plotted from the day of stem cell transplantation (day 0), with curves for each genotypic variant.

Grade 2 or more acute GVHD events during the first four weeks following HSCT were examined. The Kaplan–Meier curve in Figure 1 showed that *CYP3A5* polymorphism tended to be a factor affecting the risk of acute GVHD during the first four weeks following HSCT (36.8% for CYP3A5*1 allele carriers (*n* = 19) versus 17.6% for CYP3A5*3/*3 carriers (*n* = 17); *p* = 0.172 by log-rank test).

We also examined grade 1 or more severe AKI events during the first four weeks following HSCT. The Kaplan–Meier curve showed that there was no significant difference among CYP3A5*1 allele and CYP3A5*3/*3 genotype carriers (21.1% for CYP3A5*1 allele (*n* = 19) versus 35.3% for CYP3A5*3/*3 (*n* = 17); *p* = 0.389 by log-rank test).

### 2.5. Influence of CYP3A5*3 on Tacrolimus (C/Dpo)/(C/Div) during Switching in Route of Administration

**Figure 2:**
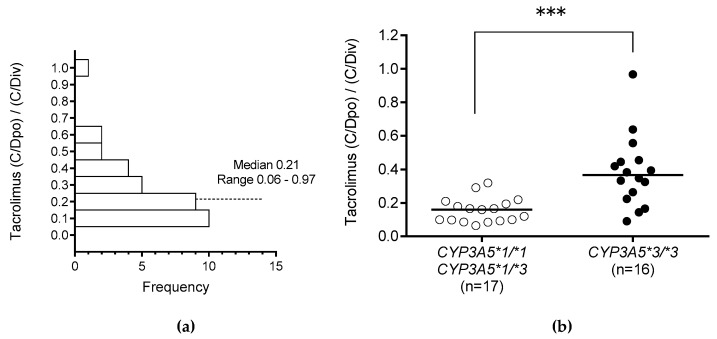
Histogram of (C/Dpo)/(C/Div) of tacrolimus (**a**) and of (C/Dpo)/(C/Div) of tacrolimus according to *CYP3A5* genotype (**b**). The C/D ratio of tacrolimus from just before the change from continuous intravenous infusion (C/Div) was compared with that from 4–7 days after the change to oral administration (C/Dpo). The bars show the median values. *** *p* < 0.001.

We examined whether the *CYP3A5* polymorphism influenced the change in the C/D ratio of tacrolimus from just before to 4–7 days after the change from continuous intravenous infusion (C/Div) to oral administration (C/Dpo). The median (C/Div) and the median (C/Dpo) of tacrolimus were calculated. The median (C/Dpo)/(C/Div) ratio was 0.21 (range, 0.06–0.97) (Figure 2a). Those with CYP3A5*3/*3 exhibited significantly higher (C/Dpo)/(C/Div) ratios than those with the CYP3A5*1 allele during switching in the route of administration (*p* < 0.001) (Figure 2b).

### 2.6. Variable Factors Influencing Tacrolimus (C/Dpo)/(C/Div) during Switching in Route of Administration

Table 5 shows the results of a univariate analysis to evaluate the potential influence of patient characteristics on increases in the (C/Dpo)/(C/Div) ratio of tacrolimus during switching in the route of administration. The (C/Dpo)/(C/Div) of tacrolimus was significantly higher in recipients with CYP3A5*3/*3 (*n* = 16, *p* < 0.001) and concomitant use of VRCZ (*n* = 7, *p* = 0.009). Concomitant use of proton pump inhibitors (PPIs), such as omeprazole or lansoprazole, which increase the C/D ratio of tacrolimus, did not significantly affect the increase in the (C/Dpo)/(C/Div) of tacrolimus (*n* = 15, *p* = 0.107). Based on a criterion of *p* < 0.10 in the univariate analysis, multiple regression analysis was carried out using CYP3A5*3/*3 and concomitant use of VRCZ as factors. The multiple regression analysis revealed that possession of the CYP3A5*3/*3 genotype and concomitant use of VRCZ were independent factors significantly contributing to increases in the (C/Dpo)/(C/Div) of tacrolimus (CYP3A5*3/*3: *p* < 0.001; VRCZ: *p* = 0.028) (Table 6).

### 2.7. Influence of Combination of Antifungal Agents and *CYP3A5* and *CYP2C19* Polymorphisms on Tacrolimus (C/Dpo)/(C/Div)

**Figure 3:**
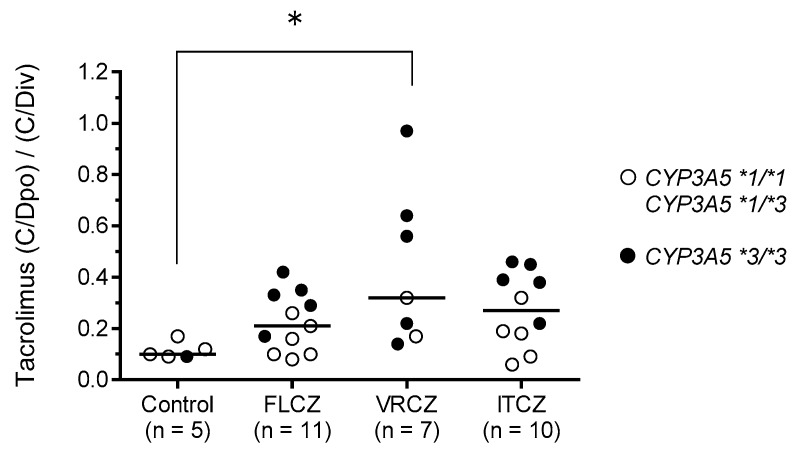
Influence of azole antifungal agents on the (C/Dpo)/(C/Div) of tacrolimus. Patients were divided into the following four groups based on the concomitant use of azole antifungal agent: Control (*n* = 5), FLCZ (*n* = 11), VRCZ (*n* = 7), and ITCZ (*n* = 10). Open circles show CYP3A5*1/*1 or CYP3A5*1/*3 genotypes (*n* = 17), and closed circles show CYP3A5*3/*3 genotype (*n* = 16). Bar shows the median value in each group. * *p* < 0.05.

The recipients were divided into the following four groups according to concomitant use of antifungal agents: Control (*n* = 5), FLCZ (*n* = 11), VRCZ (*n* = 7), and ITCZ (*n* = 10). For assessing the influence of concomitant use of antifungal agents on tacrolimus (C/Dpo)/(C/Div), we determined that the median (C/Dpo)/(C/Div) in the VRCZ group was 0.33, which was significantly higher than the median value of 0.10 observed in the control group (*p* = 0.045). Moreover, the (C/Dpo)/(C/Div) of tacrolimus tended to be higher in recipients who were CYP3A5*3/*3 carriers and concomitantly receiving azole antifungal agents (FLCZ, VRCZ, and ITCZ) (Figure 3).

Since the variation in the tacrolimus (C/Dpo)/(C/Div) was large in the VRCZ group, we examined the relationship between the (C/Dpo)/(C/Div) of tacrolimus, the trough plasma VRCZ concentration, and the *CYP3A5*, *CYP2C19*, and *POR* genotypes (Table 7). The two recipients with high (C/Dpo)/(C/Div) ratios of tacrolimus (no. 1 in Table 7: 0.97; no. 2 in Table 7: 0.70) had CYP3A5*3/*3 and were *CYP2C19* IMs. However, the two recipients with low (C/Dpo)/(C/Div) ratios of tacrolimus (no. 7 in Table 7: 0.14; no. 5 in Table 7: 0.22) had the same CYP3A5*3/*3 and *CYP2C19* IM combination. Interestingly, though, the trough plasma concentrations of VRCZ in these two recipients were 1.13 mcg/mL and 1.90 mcg/mL, respectively.

### 2.8. Assessment of Optimal Dose Conversion Ratio of Tacrolimus according to CYP3A5*3 Genotype during Switching in Route of Administration

**Figure 4:**
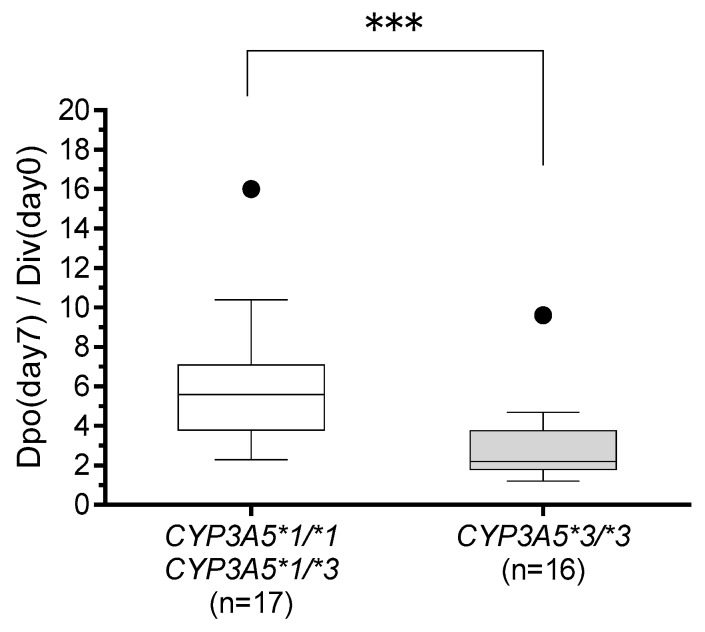
Comparison of tacrolimus dose ratio (Dpo(day7)/Div(day0)) between those with CYP3A5*1/*1 or CYP3A5*1/*3 genotypes (*n* = 17) and those with CYP3A5*3/*3 genotype (*n* = 16). The dose ratio from just before the change from continuous intravenous infusion (Div(day0)) was compared with that from seven days after the change to oral administration (Dpo(day7)). The bars show the median values, and boxes represent the 25th and 75th percentiles of the data. *** *p* < 0.001.

We examined the dosage of tacrolimus just before the change in administration route (Div(day 0)) and on day 7 after switching (Dpo(day 7)). To maintain target blood levels of tacrolimus, recipients with the CYP3A5*3/*3 genotype required a lower tacrolimus dose ratio (Dpo(day 7)/Div(day 0)) than carriers of the CYP3A5*1 allele (medians: 2.2 and 5.6, respectively; *p* < 0.001) (Figure 4).

## 3. Discussion

The main findings of this study are as follows: (1) HSCT recipients with the CYP3A5*1 allele, particularly those with at least one POR*28 allele, had a significantly reduced tacrolimus C/D ratio compared to that in HSCT recipients with POR*1/*1 during continuous intravenous infusion; (2) the CYP3A5*3/*3 genotype and the concomitant use of VRCZ are independent factors leading to an increased tacrolimus C/D ratio during switching the route of administration; and (3) conversion from intravenous to oral administration of tacrolimus at a ratio of 1:5 seemed appropriate in recipients carrying the CYP3A5*1 allele, while a lower conversion ratio, for instance 1:2–3, was appropriate in HSCT recipients with CYP3A5*3/*3.

There are several reports describing higher tacrolimus C/D ratios or trough-blood concentrations in HSCT recipients with CYP3A5*3/*3 than in those with the CYP3A5 *1 allele during continuous intravenous infusion of tacrolimus [18,19,20]. Our results are consistent with those reported previously. To our knowledge, our study demonstrates for the first time that HSCT recipients with the CYP3A5*1 allele, and those with at least one POR*28 allele, exhibit significantly lower tacrolimus C/D ratios than HSCT recipients without a POR*28 allele during continuous intravenous infusion (Table 3). This finding is consistent with respect to findings in kidney [26,35,36,37,38] and heart [39] transplant recipients. Therefore, the POR*28 allele is considered to enhance the metabolic activity of CYP3A5, rather than that of CYP3A4. In contrast, HSCT recipients with CYP3A5*3/*3 carrying one or two POR*28 alleles had significantly lower tacrolimus C/D ratios compared to those in recipients without POR*28 during the first week following HSCT. However, no significant differences were observed after excluding the influence of concomitant use of azole antifungal agents (Table 4). These results suggest that azole antifungal agents primarily inhibit the activity of CYP3A4 and that tacrolimus is mainly metabolized by CYP3A4 in subjects with CYP3A5*3/*3. Zhang et al. [40] reported similar results, in that the POR*1/*28 genotype led to a significantly lower level of tacrolimus exposure than POR28*1/*1 in CYP3A5*1/*1 carriers. Interestingly, this phenomenon disappeared in CYP3A5*3/*3 carriers identified among 71 healthy Chinese volunteers. Because the effect of the *POR* polymorphism on the metabolism of tacrolimus via CYP3A5 has not yet been fully clarified in vitro, further research will be needed.

In this study, the relationship between acute GVHD and *CYP3A5* polymorphism within four weeks after HSCT was investigated. We observed that the cumulative incidence of grade 2–4 acute GVHD tended to be higher in individuals with the CYP3A5*1 allele than in those with CYP3A5*3/*3. Khaled et al. [20] reported that eight recipients with CYP3A5*1/*1 exhibited a significantly higher cumulative rate of grade 2–4 acute GVHD than recipients with CYP3A5*1/*3 (*n* = 40) and CYP3A5*3/*3 (*n* = 122) within 100 days after HSCT. Yamashita et al. [21] reported that recipients with the CYP3A5*1 allele (*n* = 11) exhibited a significantly higher cumulative rate of grade 3–4 acute GVHD than those with CYP3A5*3/*3 (*n* = 13) within 100 days after HSCT. In this study, although there were three CYP3A5*1/*1 recipients (data not shown), grade 3–4 acute GVHD occurred in only two recipients (data not shown). Thus, although a low C/D ratio or blood concentration of tacrolimus may be associated with the CYP3A5*1 allele rather than with the CYP3A5*3/*3 genotype, statistical analysis could not be performed due to the small number of cases; studies including a large number of subjects are therefore needed.

The influence of *CYP3A5* polymorphism on the tacrolimus (C/Dpo)/(C/Div) ratio when switching the drug administration route from continuous intravenous infusion to oral administration was significantly higher in those with CYP3A5*3/*3 than in those with the CYP3A5*1 allele (Figure 2B). This result suggests that CYP3A5 is expressed in the liver and small intestine, and indeed, CYP3A5 has been shown to play a very important role in the small intestine [9,41,42,43,44]. In this study, multiple regression analysis revealed that CYP3A5*3/*3 was one of the independent factors contributing to a significant increase in (C/Dpo)/(C/Div). Moreover, the (C/Dpo)/(C/Div) of tacrolimus tended to be higher in recipients with CYP3A5*3/*3 and those receiving azole antifungal agents than in those with the CYP3A5*1 allele (Figure 3). Yamashita et al. [21] reported that among recipients undergoing concomitant use of azole antifungal agents, the trough concentration of tacrolimus was higher in recipients with CYP3A5*3/*3 than in those with the CYP3A5*1 allele, although the daily doses of once-daily modified-release tacrolimus formulations in recipients with CYP3A5*3/*3 were significantly lower than in those with the CYP3A5*1 allele. Azole antifungal agents have a stronger inhibitory effect on CYP3A4 activity than on CYP3A5 in the small intestine [29,31], and tacrolimus is metabolized by CYP3A4 in recipients expressing CYP3A5*3/*3. As a result of CYP3A4 inhibition by azole antifungal agents in the small intestine, the (C/Dpo)/(C/Div) of tacrolimus tends to be higher in CYP3A5*3/*3 recipients when azole antifungal agents are co-administered. Therefore, when switching the route of administration, it is important to consider the combination of the *CYP3A5* genotype and the concomitant use of azole antifungal agents in addition to the therapeutic drug monitoring of tacrolimus. Tacrolimus is often concomitantly administered with PPIs, giving rise to drug interaction issues. Interactions between tacrolimus and PPIs are affected by a combination of the CYP2C19 and CYP3A5 polymorphisms [45]. In this study, concomitant administration of omeprazole or lansoprazole, both of which increase the C/D ratio of tacrolimus, did not significantly impact the increase in the (C/Dpo)/(C/Div) of tacrolimus. Since the number of cases included in this study was small, this may need to be confirmed in a larger number of cases.

In this study, the concomitant use of VRCZ was identified as another independent factor that significantly increased the (C/Dpo)/(C/Div) of tacrolimus. Imamura et al. [34] reported that *CYP2C19* PMs and IMs achieve 4- and 2-fold higher VRCZ exposures (areas under the curve), respectively, than that achieved by EMs. Iwamoto et al. [19] reported that the dose of tacrolimus in continuous intravenous infusion varies depending on the combination of *CYP3A5* and *CYP2C19* genotypes in HSCT recipients treated with VRCZ. In this study, as we analyzed only seven recipients with VRCZ use, we could not statistically analyze the effects of the combination of *CYP3A5* and *CYP2C19* genotypes on the (C/Dpo)/(C/Div) of tacrolimus (Table 7). Both the two recipients with high (C/Dpo)/(C/Div) of tacrolimus and those with low (C/Dpo)/(C/Div) were of the CYP3A5*3/*3 and *CYP2C19* IM genotypes. However, the trough plasma concentrations of VRCZ in the latter two recipients were near the lower limit of the recommended target trough value of >1–2 mcg/mL [46]. These findings can be explained on the basis of in vitro human liver microsome experiments that demonstrated that the magnitude of the inhibition of tacrolimus metabolism by VRCZ is concentration dependent [47]. Therefore, our results suggest the importance of not only the combination of the *CYP3A5* and *CYP2C19* genotypes, but also the plasma concentration of VRCZ.

According to the guidelines for GVHD from the Japanese Society for Hematopoietic Cell Transplantation, a 3- to 4-fold higher dosage is recommended when switching from continuous intravenous infusion to oral administration of tacrolimus with HSCT. We have previously reported that switching from intravenous to oral administration at a 1:5 ratio seems appropriate and that a lower conversion ratio, such as 1:3, is appropriate for patients taking oral ITCZ or VRCZ [48]. However, these recommendations did not consider the genetic polymorphisms, especially of *CYP3A5*, that affect the pharmacokinetics of tacrolimus. In this study, multiple regression analysis revealed the CYP3A5*3/*3 genotype as an independent factor significantly increasing (C/Dpo)/(C/Div). Our result indicates that switching from intravenous to oral administration of tacrolimus at a ratio of 1:5 in recipients with the CYP3A5*1 allele is seemingly appropriate, while a lower conversion ratio such as 1:2–3 may be suitable in recipients with CYP3A5*3/*3 (Figure 4). Moreover, the concomitant use of VRCZ is also a significant independent factor leading to increased tacrolimus (C/Dpo)/(C/Div), and large individual variation was observed in the (C/Dpo)/(C/Div) of tacrolimus. Therefore, conversion is recommended under close medical supervision, and readjustment of the tacrolimus dose should be done in consideration of its blood level.

This study had several limitations. First, this study had a small sample size of only 36 cases and was conducted at a single facility. Second, the initial dosage and the conversion ratio of tacrolimus between intravenous and oral administration were not standardized because this was an observational study. Regardless of the limitations described above, our results indicate that the *CYP3A5*, *POR*, and *CYP2C19* polymorphisms are useful for the dosage adjustment of tacrolimus in association with estimation of the systemic pharmacokinetics of tacrolimus. It may be important to adjust the target level of tacrolimus both after the initial post-transplantation period on the basis of the *CYP3A5* genotype in combination with the *POR* genotype and when switching the administration route based on the *CYP3A5* genotype and combination with the *CYP2C19* genotype in recipients receiving VRCZ.

## 4. Materials and Methods

### 4.1. Patients and Tacrolimus Administration

Between August 2009 to July 2018, we enrolled 36 Japanese recipients with hematological disorders who underwent first allogeneic HSCT receiving tacrolimus for prophylaxis of acute GVHD at Kyushu University Hospital. One recipient was excluded from this study because of a history of living-donor liver transplantation.

The initial dose of tacrolimus continuous intravenous infusion was 0.02–0.03 mg/kg/day starting on the day prior to HSCT. When recipients could tolerate oral administration, the route of administration of tacrolimus was switched to an oral dose 2–4 times greater than the continuous intravenous dose, which was given in two divided doses. Intravenous infusion was stopped just before the first oral administration of medication. The target tacrolimus blood concentrations were 10–15 ng/mL with continuous intravenous infusion and 8–12 ng/mL after switching to oral administration. The dose of tacrolimus was modified at the discretion of each physician.

This study was conducted in accordance with the Declaration of Helsinki and its amendments and was approved by the Ethics Committee of Kyushu University Graduate School and Faculty of Medicine (approval no. 652–01, approved on 5 July 2018).

### 4.2. Clinical Samples, Data Collection, and Study Endpoints

The tacrolimus blood concentration was measured using either an antibody-conjugated magnetic immunoassay on the Dimension Xpand system (Siemens Japan K.K., Tokyo, Japan; accessed during August 2009–December 2010) or a chemiluminescent immunoassay on the Architect-i1000 system (Abbott Japan, Tokyo, Japan; accessed during December 2010 to July 2018). The equivalence of the data obtained using these two methods was validated (data not shown). Tacrolimus blood concentration was measured almost every day during continuous intravenous infusion and three times a week after switching to oral administration. Plasma VRCZ concentration was measured by outsourcing (SRL, Inc., Tokyo, Japan or LSI Medience Corporation, Tokyo, Japan) and the results obtained by ultra-performance liquid chromatography or ultra-performance liquid chromatography-tandem mass spectrometry.

All data were retrospectively collected from the electronic medical record system. The trough-blood concentration and dose of tacrolimus were recorded for all recipients for the first 100 days. The pharmacokinetics of tacrolimus were evaluated by the blood trough concentration/dose (C/D, (ng/mL)/(mg/day)) ratio. The evaluation period was three weeks after HSCT at the time of continuous intravenous infusion. The C/D ratio just before the change from continuous intravenous infusion (C/Div) was compared with that from 4–7 days after the change to oral administration (C/Dpo), when the increase in the tacrolimus blood concentration was stabilized.

The primary endpoint of this study was an evaluation of the utility of the *CYP3A5* genotype in determining the tacrolimus C/D ratio during continuous intravenous infusion and after switching the route of administration in HSCT recipients. Secondary endpoints included the influence of combinations of *POR* and *CYP3A5* genotypes or *CYP2C19* and *CYP3A5* genotypes on the tacrolimus C/D ratio, the relationship between the *CYP3A5* genotype and acute GVHD or acute kidney injury (AKI) during the first 4 weeks following HSCT, factors affecting the C/D ratio of tacrolimus following switching of the route of administration, and the determination of the appropriate dose conversion ratio of tacrolimus when switching the route of administration. Acute GVHD was graded as described previously [49]. AKI was defined according to common terminology criteria for adverse events (CTCAE) Ver4.0. Baseline serum creatinine (Scr) was the value obtained before the start of conditioning therapy. With regard to drug interactions between the tacrolimus C/D ratio and concomitant use of azole antifungal agents, we divided antifungal agent use into four groups—without azole antifungal agent (control), FLCZ, VRCZ, and ITCZ—according to the ability of the agents to inhibit the CYP3A4 enzyme system [29,30,31]. In subgroup analyses restricted to recipients without concomitant use of azole antifungal agents, values of the tacrolimus C/D ratio obtained at the following time points were excluded based on the half-life of the relevant antifungal: During administration and within 5 days of discontinuation of FLCZ or ITCZ, and during administration and within 3 days of discontinuation of VRCZ.

### 4.3. Genotyping of *CYP3A5*, *POR*, and *CYP2C19*

Genomic DNA was extracted from buccal mucosa samples obtained after HSCT or peripheral blood or bone marrow samples obtained before HSCT using the QIAamp mini kit (Qiagen, Hilden, Germany) according to the manufacturer’s instructions. The CYP3A5*3, POR*28, CYP2C19*2, and CYP2C19*3 SNPs were detected using a previously described real-time polymerase chain reaction method with a Light-Cycler (Roche, Mannheim, Germany) [50,51]. Based on the *CYP3A5* genotype, recipients were classified into two groups: Those with the CYP3A5*1 allele (CYP3A5*1/*1 or CYP3A5*1/*3) and those with the CYP3A5*3/*3 genotype. Based on the *POR* genotype, recipients were classified into two groups: Those with the POR*28 allele (POR*1/*28 or POR*28/*28) and those with the POR*1/*1 genotype. Based on the *CYP2C19* genotype, recipients were classified into three groups: Extensive metabolizers (EM; CYP2C19*1/*1), intermediate metabolizers (IM; CYP2C19*1/*2 or *1/*3), and poor metabolizers (PM; CYP2C19*2/*2 or *3/*3). The results of *CYP3A5*, *POR*, and *CYP2C19* genotyping were not used to adjust tacrolimus dosage.

### 4.4. Statistical Analysis

Differences between two groups were evaluated using the chi-square test or Fisher’s exact test for categorical variables and the Mann–Whitney *U*-test for continuous variables. The Kruskal–Wallis test followed by Dunn’s multiple comparison test was used to evaluate comparisons among three or more groups. The probabilities of acute GVHD and AKI were estimated using the Kaplan–Meier method and were compared using log-rank analysis. These statistical analyses were carried out using GraphPad Prism version 6 (GraphPad Software, San Diego, CA, USA). Values with borderline significance (*p* < 0.10) were subjected to multiple regression analyses with backward selection. Univariate and multivariate logistic regression analyses were carried out using JMP 13 (SAS Institute Inc., Cary, NC, USA). Results with *p-*values of < 0.05 were considered statistically significant.

## Figures and Tables

**Table 1:** Demographics of hematopoietic stem cell transplantation (HSCT) recipients according to *CYP3A5* genotype (*n* = 36).

Characteristics	CYP3A5 *1/*1 or *1/*3 (*n* = 19)	CYP3A5 *3/*3 (*n* = 17)	*p* Value
Age, Median, year (range)	55 (34–69)	55 (17–67)	0.894
Gender, Male/Female, no.	11/8	10/7	1.000
Body weight, Median, kg (range)	63.1 (42.0–98.9)	59.3 (43.6–74.1)	0.367
POR*28 genotype, no.			0.335
*1/*1	10	6	
*1/*28 or *28/*28	9	11	
***CYP2C19* genotype, no.**			0.230
*1/*1	5	5	
*1/*2 or *1/*3	11	12	
*2/*2 or *3/*3	3	0	
**Diagnosis, no.**			0.236
AML/MDS	13	7	
ALL	1	2	
CML	1	1	
Lymphoma	3	7	
AA	1	0	
**Donor type and stem cell, no.**			0.667
Unrelated, bone marrow	10	9	
Unrelated, peripheral blood	1	1	
Unrelated, cord blood	3	4	
Related, peripheral blood	0	1	
Related, haplo-peripheral blood	5	2	
**HLA, no.**			0.281
Full match	4	7	
Mismatch	15	10	
**Conditioning regimen, no.**			1.000
Myeloablative	6	6	
Reduced intensity	13	11	
**GVHD prophylaxis, no.**			0.157
MTX	11	13	
MMF	3	4	
mPSL	3	0	
MMF + CY	2	0	
**Concomitant antifungal agents, no.**			0.422
CPFG or MCFG	15	10	
FLCZ	3	5	
VRCZ	1	2	
AST, Median, U/L (range)	22 (12–57)	21 (10–32)	0.567
ALT, Median, U/L (range)	29 (10–71)	20 (8–54)	0.101
T-Bil, Median, mg/dL (range)	0.5 (0.3–1.4)	0.4 (0.3–1.0)	0.077
Scr, Median, mg/dL (range)	0.67 (0.52–1.38)	0.63 (0.42–1.06)	0.182

AML indicates acute myeloid leukemia; ALL, acute lymphoblastic leukemia, CML, chronic myeloid leukemia; AA, aplastic anemia; MTX, methotrexate; MMF, mycophenolate mofetil; mPSL, methylprednisolone; CY, cyclophosphamide; CPFG, aspofungin; MCFG. micafungin; FLCZ, fluconazole; VRCZ, voriconazole; AST, aspartate aminotransferase; ALT, alanine aminotransferase; T-Bil, total bilirubin; Scr, serum creatinine.

**Table 2:** Day +1 to day +21 median tacrolimus concentration/dose (C/D) ratio according to *CYP3A5* genotype (*n* = 36).

Days	*CYP3A5* Genotype								
	CYP3A5 *1/*1 or *1/*3				CYP3A5 *3/*3				*p* Value
	C/D Ratio, Median	Range	*n* ^a^ (Sample ^b^)		C/D Ratio, Median	Range	*n* ^a^ (Sample ^b^)		
All patients									
Day 1–7	11.5	4.7–18.9	19 (117) ^c^		12.7	4.8–22.7	17 (119)		0.032
Day 8–14	10.0	3.4–15.5	19 (133)		11.2	4.2–26.2	17 (118)		0.001
Day 15–21	11.0	4.8–20.3	19 (131)		10.8	4.9–19.3	17 (118)		0.860
Without concomitant azoles ^d^									
Day 1–7	11.5	4.7–18.9	15 (91)		10.6	4.8–21.0	10 (70)		0.398
Day 8–14	9.9	3.4–15.5	16 (107)		9.9	4.8–26.2	13 (77)		0.048
Day 15–21	10.5	4.8–20.3	17 (117)		10.3	4.9–18.4	15 (100)		0.569

C/D indicates concentration/dose. ^a^ Number of subjects. ^b^ Number of sample. ^c^ Two patients started tacrolimus on day 5 after HSCT because of HLA-haploidentical hematopoietic stem cell transplantation. ^d^ Values of tacrolimus C/D ratio obtained at the following time course were excluded based on their half-life: Administration period and within five days of discontinuation in fluconazole or itraconazole, and administration period and within three days of discontinuation in voriconazole.

**Table 3:** Day +1 to day +21 median tacrolimus C/D ratio of CYP3A5*1/*1 and CYP3A5*1/*3 carriers according to *POR* genotype combinations (*n* = 19).

Days	CYP3A5 *1/*1 or *1/*3 (*n* = 19)								
	POR *1/*1				POR *1/*28 or *28/*28				*p* Value
	C/D Ratio, Median	Range	*n* ^a^ (Sample ^b^)		C/D Ratio, Median	Range	*n* ^a^ (Sample ^b^)		
All patients									
Day 1–7	12.3	5.9–18.9	10 (67)		10.3	4.7–16.8	9 (50)		<0.001
Day 8–14	10.6	4.5–15.5	10 (70)		8.9	3.4–15.0	9 (63)		0.030
Day 15–21	12.4	4.8–20.3	10 (68)		9.3	5.1–18.7	9 (63)		<0.001
Without concomitant azoles ^c^									
Day 1–7	13.0	5.9–18.9	7 (48)		10.1	4.7–16.8	8 (43)		0.001
Day 8–14	10.1	4.5–15.5	8 (51)		9.4	3.4–15.0	8 (56)		0.418
Day 15–21	12.25	4.8–20.3	8 (54)		9.3	5.1–18.7	9 (63)		<0.001

C/D indicates concentration/dose. ^a^ Number of subjects. ^b^ Number of sample. ^c^ Values of tacrolimus C/D ratio obtained at the following time course were excluded based on their half-life: Administration period and within five days of discontinuation in fluconazole or itraconazole, and administration period and within three days of discontinuation in voriconazole.

**Table 4:** Day +1 to day +21 median tacrolimus C/D ratio of CYP3A5*3/*3 carriers according to *POR* genotype combinations (*n* = 17).

Days	CYP3A5 *3/*3 (*n* = 17)								
	POR *1/*1				POR *1/*28 or *28/*28				*p* Value
	C/D Ratio, Median	Range	*n* ^a^ (Sample ^b^)		C/D Ratio, Median	Range	*n* ^a^ (Sample ^b^)		
All patients									
Day 1–7	16.05	7.0–22.7	6 (42)		11.6	4.8–22.0	11 (77)		0.003
Day 8–14	11.5	6.6–26.2	6 (42)		10.5	4.2–20.0	11 (76)		0.178
Day 15–21	11.25	6.9–18.0	6 (42)		10.3	4.9–19.3	11 (76)		0.350
Without concomitant azoles ^c^									
Day 1–7	9.8	7.0–21.0	3 (21)		10.8	4.8–18.9	7 (49)		0.686
Day 8–14	10.45	7.9–26.2	4 (22)		9.1	4.8–20.0	9 (55)		0.159
Day 15–21	10.5	6.9–18.0	5 (31)		10.2	4.9–18.4	10 (69)		0.517

C/D indicates concentration/dose. ^a^ Number of subjects. ^b^ Number of sample. ^c^ Values of tacrolimus C/D ratio obtained at the following time course were excluded based on their half-life: Administration period and within five days of discontinuation in fluconazole or itraconazole, and administration period and within three days of discontinuation in voriconazole.

**Table 5:** Univariate logistic regression analysis of variables associated with an increase in tacrolimus (C/Dpo)/(C/Div) (*n* = 33).

Variables	(C/D)po/(C/D)iv	
	*n*	*p* Value
Age (years)	―	0.294
Male	19	0.829
CYP3A5 *3/*3	16	<0.001
POR *1/*1	14	0.700
*CYP2C19 IM or PM*	22	0.960
Concomitant FLCZ	11	0.392
Concomitant VRCZ	7	0.009
Concomitant ITCZ	10	0.874
AST Grade1 or more	4	0.131
ALT Grade1 or more	13	0.513
T-Bil Grade1 or more	1	0.365

FLCZ indicates fluconazole; VRCZ, voriconazole; ITCZ, itraconazole; AST, aspartate aminotransferase; ALT, alanine aminotransferase; T-Bil, total bilirubin.

**Table 6:** Multiple logistic regression analysis of variables associated with an increase in tacrolimus (C/Dpo)/(C/Div) (*n* = 33).

Variables	(C/D)po/(C/D)iv	*p* Value
CYP3A5 *3/*3	0.31 + 0.10 (CYP3A5 *3/*3 Genotype) + 0.07 (Concomitant VRCZ)	<0.001
Concomitant VRCZ		0.028

VRCZ indicates voriconazole. Multivariate analysis with variables *p* < 0.1 in univariate analysis.

**Table 7:** Tacrolimus (C/Dpo)/(C/Div) and variables associated with *CYP3A5*, *CYP2C19*, and *POR* genotypes in recipients receiving VRCZ (*n* = 7).

No.	Tacrolimus (C/D)po/(C/D)iv	Genotypes				VRCZ		
		*CYP3A5*	*CYP2C19*	*POR*		Dose/Day (mg)	Route of administration	Plasma Trough Concentration ^a^ (mcg/mL)
1	0.96	*3/*3	IMs	*1/*1		300	Oral	1.84
2	0.70	*3/*3	IMs	*1/*28		600	Oral	4.25
3	0.56	*3/*3	EMs	*1/*1		400	Oral	3.07
4	0.33	*1/*3	EMs	*1/*28		400	Oral	0.82
5	0.22	*3/*3	IMs	*1/*28		400	Oral	1.90
6	0.17	*1/*3	PMs	*1/*28		400	Oral	2.72
7	0.14	*3/*3	IMs	*1/*28		400	Oral	1.13

VRCZ indicates voriconazole; CYP2C19 EMs, CYP2C19 *1/*1; CYP2C19 IMs, CYP2C19 *1/*2 or *1/*3; CYP2C19 PMs, CYP2C19 *2/*2 or *3/*3. ^a^ Plasma Trough VRCZ concentrations were measured from −1 to 9 days after switching the route of tacrolimus.
